# Octadecaneuropeptide (ODN) Induces N2a Cells Differentiation through a PKA/PLC/PKC/MEK/ERK-Dependent Pathway: Incidence on Peroxisome, Mitochondria, and Lipid Profiles

**DOI:** 10.3390/molecules24183310

**Published:** 2019-09-11

**Authors:** Amira Namsi, Thomas Nury, Amira. S. Khan, Jérôme Leprince, David Vaudry, Claudio Caccia, Valerio Leoni, Atanas G. Atanasov, Marie-Christine Tonon, Olfa Masmoudi-Kouki, Gérard Lizard

**Affiliations:** 1 Team Bio-PeroxIL, Biochemistry of the Peroxisome, Inflammation and Lipid Metabolism (EA7270)/University Bourgogne Franche-Comté (UBFC)/Inserm, 21000 Dijon, France; amira.namsi@gmail.com (A.N.); thomas.nury@u-bourgogne.fr (T.N.); 2 Faculty of Science of Tunis, University Tunis El Manar, LR18ES03, Laboratory of Neurophysiology, Cellular Physiopathology and Biomolecules Valorisation, Tunis 2092, Tunisia; 3 Physiology of Nutrition & Toxicology (NUTox), Inserm U1231, University UBFC, 21000 Dijon, France; amira.khan@u-bourgogne.fr; 4 UNIROUEN, Inserm U1239, Laboratory of Neuronal and Neuroendocrine Communication and Differentiation, Normandie University, 76000 Rouen, France; jerome.leprince@univ-rouen.fr (J.L.); david.vaudry@univ-rouen.fr (D.V.); marie-christine.tonon@univ-rouen.fr (M.-C.T.); 5 UNIROUEN, Regional Cell Imaging Platform of Normandy (PRIMACEN), Normandie University, 76000 Rouen, France; 6 Laboratory of Medical Genetics and Neurogenetics, Foundation IRCCS Istituto Neurologico Carlo Besta, 20100 Milan, Italy; claudio.caccia@istituto-besta.it; 7 Laboratory of Clinical Chemistry, Hospital of Varese, ASST-Settelaghi, 20100 Milan, Italy; valerioleoni@hotmail.com; 8 Institute of Genetics and Animal Breeding of the Polish Academy of Sciences, Jastrzebiec, 05-552 Magdalenka, Poland; atanas.atanasov@univie.ac.at; 9 Department of Pharmacognosy, University of Vienna, 1010 Vienna, Austria; 10 Institute of Neurobiology, Bulgarian Academy of Sciences, 23 Acad. G. Bonchev str., 1113 Sofia, Bulgaria

**Keywords:** octadecaneuropeptide (ODN), N2a cells, neuronal differentiation, mitochondria, peroxisome, fatty acids, cholesterol, cholesterol precursors

## Abstract

Neurodegenerative diseases are characterized by oxidative stress, mitochondrial damage, and death of neuronal cells. To counteract such damage and to favor neurogenesis, neurotrophic factors could be used as therapeutic agents. Octadecaneuropeptide (ODN), produced by astrocytes, is a potent neuroprotective agent. In N2a cells, we studied the ability of ODN to promote neuronal differentiation. This parameter was evaluated by phase contrast microscopy, staining with crystal violet, cresyl blue, and Sulforhodamine 101. The effect of ODN on cell viability and mitochondrial activity was determined with fluorescein diacetate and DiOC_6_(3), respectively. The impact of ODN on the topography of mitochondria and peroxisomes, two tightly connected organelles involved in nerve cell functions and lipid metabolism, was evaluated by transmission electron microscopy and fluorescence microscopy: detection of mitochondria with MitoTracker Red, and peroxisome with an antibody directed against the ABCD3 peroxisomal transporter. The profiles in fatty acids, cholesterol, and cholesterol precursors were determined by gas chromatography, in some cases coupled with mass spectrometry. Treatment of N2a cells with ODN (10^−14^ M, 48 h) induces neurite outgrowth. ODN-induced neuronal differentiation was associated with modification of topographical distribution of mitochondria and peroxisomes throughout the neurites and did not affect cell viability and mitochondrial activity. The inhibition of ODN-induced N2a differentiation with H89, U73122, chelerythrine and U0126 supports the activation of a PKA/PLC/PKC/MEK/ERK-dependent signaling pathway. Although there is no difference in fatty acid profile between control and ODN-treated cells, the level of cholesterol and some of its precursors (lanosterol, desmosterol, lathosterol) was increased in ODN-treated cells. The ability of ODN to induce neuronal differentiation without cytotoxicity reinforces the interest for this neuropeptide with neurotrophic properties to overcome nerve cell damage in major neurodegenerative diseases.

## 1. Introduction

Neurodegenerative diseases such as Alzheimer’s disease (AD), Parkinson’s disease (PD), and multiple sclerosis (MS) are characterized by neuronal cell death caused in part by an inflammatory process, mitochondrial alterations, and an elevation of oxidative stress [1,2,3,4]. As AD, PD, and MS have a significant societal and financial impact, and due to the lack of drugs to treat these diseases (mainly AD and PD), it is crucial to identify new therapies for effective treatments [5,6]. Among the various possible therapeutic approaches, we attempt to identify molecules which can prevent oxidative damages and mitochondrial dysfunctions leading to cell death. An additional challenge is to identify molecules which are also able to favor neuronal regeneration, and which can permit the repair of injured brain regions. In this context, polyphenols are interesting natural molecules for the development of therapeutic strategies to cure neurodegenerative diseases because they cross the blood brain barrier and exhibit anti-apoptotic, anti-inflammatory, and anti-oxidant activities [7,8]. Polyphenols such as resveratrol and apigenin have been shown to promote neuronal survival and differentiation in murine N2a cells [9]. Polyphenols stimulate neuritogenesis (outgrowth of dendrites and/or axons) during neuronal cell differentiation, indicating that they exert neurotrophic activities [10,11]. The neuroprotective activities of these polyphenols trigger the neurotrophic (CREB: cAMP response element-binding protein) and antioxidant (Nrf-2—ARE: nuclear factor erythroid 2—related factor 2 and antioxidant response element) defense systems in neurons [12]. Similar neuroprotective properties were observed with the brain derived neurotrophic factor (BDNF) [12,13]. Therefore, neurotrophic peptidic factors could be used to stimulate neurogenesis and prevent neuronal loss associated with neurodegeneration.

In this context, ODN, which is exclusively produced by astroglial cells in the central nervous system of mammals [14], and which stimulates neurogenesis in adult mouse brains [15], along with nerve regeneration [16], could be a promising factor. ODN is a gliopeptide generated through proteolytic cleavage of an 86-amino acid precursor called diazepam-binding inhibitor (DBI), and is able to prevent both neuron and astroglia cell death induced by several neurotoxic substances including hydrogen peroxide (H_2_O_2_) [17,18] and 6-hydroxydopamine (6-OHDA) [19,20]. The neuroprotective activity of ODN has also been observed in vivo, in the MPTP model of Parkinson’s disease [21]. For instance, ODN prevents oxidative stress-induced intracellular reactive oxygen species (ROS) accumulation, glutathione depletion, and a decrease of antioxidant enzyme expression [22]. Furthermore, ODN is able to counteract oxidative stress-induced increase of the mitochondrial respiration rate, mitochondrial dysfunctions, and stimulation of caspase-3 activation [19,23]. Altogether, these data indicate that ODN could act as an endogenous neurotrophic factor regulating proliferation and/or survival of neuronal cells.

Although the intracellular mechanisms mediating the cytoprotective effects of ODN on neurons and glial cells are clearly established [18,19,23], the signaling pathways and the mechanisms implicated in the effect of ODN on neuronal cell differentiation, as well as its simultaneous impact on organelles have not been characterized so far. To date, the important contribution of the peroxisome in nerve cell functions is based on clinical evidence. Peroxisomal disorders observed in rare genetic diseases are grouped as peroxysomopathies, which include peroxisomal biogenesis disorders (such as Zellweger syndrome) and single and/or multiple peroxisomal enzyme deficiencies (X-linked adrenoleukodystrophy (X-ALD), acyl CoA-oxidase-1 (ACOX-1) deficiency, and D bifunctional protein (MFP2) deficiency) [24]. Currently, it is also well established that peroxisomes and mitochondria are closely related organelles, and that the activity of one can influence the activity of the other [25,26,27]. So, it is important to simultaneously consider the peroxisome and the mitochondria in major biological processes such as neurodegeneration, neurogenesis, and neuronal differentiation [28,29]. Indeed, in peroxisome deficient mice, morphological and functional abnormalities of mitochondria were observed [30,31,32,33]. The inactivation of ABCD1, a peroxisomal very long chain fatty acid (VLCFA) transporter causative for X-ALD, induces oxidative damage to mitochondrial proteins and impairs oxidative phosphorylation in the spinal cord of mice [34]. Furthermore, the phototoxic effects of the genetically encoded Killer Red protein, which permitted precise analysis of the interplay between peroxisomes and cellular oxidative stress, also induced mitochondria-mediated cell death [35]. Whereas many studies highlight dysfunction of mitochondria in major neurodegenerative diseases (AD, MS, PD, and amyotophic lateral sclerosis (ALS)) [36,37,38,39,40], the part taken by the peroxisome in these diseases is not well known, whereas there is evidence that peroxisome abnormalities also occur in patients with AD and MS [41,42,43]. These arguments are based on the accumulation of VLCFA (C22 and higher), modifications of the plasmalogen profile in the plasma and red blood cells, and of peroxisomal density in brain lesions [44,45,46]. Since mitochondria and peroxisomes are involved in the metabolism of medium and long chain fatty acids, and VLCFA [47,48], respectively, as well as in the metabolism of cholesterol, it is questionable whether neuronal differentiation could modify the profiles of fatty acids, cholesterol, and cholesterol precursors. The interest in addressing these lipid profiles is also based on previous studies showing metabolic reprogramming during neuronal differentiation [49].

The aim of the present study was to investigate the potential effect of ODN on neural differentiation and to delineate its simultaneous impact on mitochondria and peroxisomes. The signaling pathway implicated in ODN-induced differentiation was also studied. N2a cells were used as a model to evaluate the impact of ODN on neuronal differentiation, as well as its subsequent effects on the cell distribution of mitochondria, peroxisome, and on lipid metabolism. These cells were chosen for the following reasons: (1) they express the pituitary adenylate cyclase-activating polypeptide type I receptor also known as PAC1, which is a member of the G-protein coupled receptor (GPCR) superfamily including the metabotropic receptors which bind ODN [23,50]; (2) they also have the ability to bind the pituitary adenylate cyclase-activating polypeptide (PACAP), which is widely distributed in the brain and peripheral organs, which displays high affinity for the PAC1 receptor [51,52], and which induces PC12 cell differentiation [53,54]; (3) they have the ability to differentiate in mature neurons and to form neurites [9]. 

In the present study, we show that ODN induces neuronal differentiation in N2a cells supporting that this molecule, which also has cytoprotective properties, can be considered as a neurotrophin [55]. 

## 2. Materials and Methods

### 2.1. Cell Culture and Treatments

Mouse neuro-2a (N2a) neuroblastoma cells (ATCC^®^ CCL-131™) were purchased from the American Type Culture Collection (ATCC, Rockville, MD, USA). N2a cells were plated at a density of 3.4 × 10^4^ cells/cm^2^; they were cultured in Dulbecco’s modified Eagle medium (DMEM, Lonza, Amboise, France) with high glucose (4.5 g/L), glutamine, and sodium pyruvate (Dominique Dutscher, Brumath, France) supplemented with 10% (*v/v*) fetal bovine serum (FBS, Pan Biotech, Aidenbach, Germany) containing 1% (*v/v*) antibiotics (100 U/mL penicillin, 100 mg/mL streptomycin) (Pan Biotech). Human neuroblastoma SK-N-BE cells purchased from the ATCC were plated at a density of 1 × 10^4^ cells/cm^2^; they were cultured in glucose-rich DMEM medium (4.5 g/L) with glutamine and sodium pyruvate containing 10% FBS. The FBS used was always heat inactivated. N2a and SK-N-BE cells were incubated at 37 °C in a 5% CO_2_ humidified atmosphere, and passaged twice a week. 

To evaluate the differentiating properties of ODN, N2a cells were cultured at a density of 1.2 × 10^5^ per well in 6-well plates (FALCON, Becton Dickinson, NJ, USA) or in tissue culture dishes (35 × 10 mm, FALCON) containing 1 mL of culture medium with 10% FBS. The differentiating properties of ODN were also evaluated on SK-N-BE cells. To this end, SK-N-BE cells were cultured at a density of 5 × 10^3^ per well in 6-well plates (FALCON) or in tissue culture dishes (35 × 10 mm, FALCON) containing 1 mL of culture medium with 10% FBS. After 24 h of culture, the culture medium of N2a and SK-N-BE cells was removed, and the cells were cultured for 48 h in the absence or presence of octadecaneuropeptide (ODN: 10^−16^ to 10^−12^ M) in culture medium without (0%) or with 10% FBS. ODN (QATVGDVNTDRPGLLDLK) was synthesized by using the standard Fmoc procedure [56]. 

### 2.2. Evaluation of Neuronal Differentiation With Morphological Criteria

N2a cells (morphologically resembling neuroblasts) have the ability to differentiate in young immature and mature neurons with neurites (evocating dendrites and/or axons). Morphological criteria were used to evaluate neuronal differentiation on N2a cells culture. These criteria were previously defined on N2a cells with the use of ODN, as well as of polyphenols (resveratrol, apigenin), which are also capable of inducing neuronal differentiation on N2a cells [9]. In control cells, whereas mainly neuroblasts were present (cells without neurites: undifferentiated cells), several cells with several neurites of different length (differentiated cells include: cells with several neurites of average length (5–10 µm); cells with several neurites (including one or more neurites of important length (>10 µm) evocating dendrites and/or axons without or with neurites (5–10 µm)) (Supplementary Figure S1). Thus, after 24 h of culture in 6-well plates in DMEM with 10% FBS, the culture medium was removed and N2a cells were cultured for 48 h in the presence of ODN (10^−16^ to 10^−12^ M) without or with 10% FBS. Cells were either directly observed by phase contrast microscopy [9] or brightfield microscopy following staining with crystal violet [57]. The morphological criteria used to evaluate neuronal differentiation are the same by phase contrast microscopy and after staining with crystal violet. In addition, cresyl blue staining procedure, which may be associated with a crystal violet counterstaining, was used to evaluate neuronal differentiation. Cresyl blue is a conventional histocytological staining method which permits to reveal Nissl bodies in neural cells ((https://pathologycenter.jp/method-e/nissl.html; 2019). Nissl bodies consist of a large number of rough endoplasmic reticulum and free ribosomes. The increased density of Nissl bodies associated with neuronal differentiation is characterized by a pronounced blue staining in the cytoplasm of differentiated cells. Briefly, N2a cells cultured in Lab Tek chambers were stained for 30 min by cresyl blue, fixed with 4% filtered paraformaldehyde (5 min, 22 °C), and washed 3 times with PBS. The Nissl bodies were observed by brightfield microscopy using a right Zeiss microscope (Skope.A1, Jena, Germany), at a ×63 magnification (Objective: 63 × 1.25 oil, EC plan NEOFLUAR, ref: 420480-9900) and digitalized images were obtained with a Zeiss camera (Axiocam). The observations (phase contrast microscopy; crystal violet) were performed with an inverted Zeiss microscope (Primovert) at a ×20 magnification (Objective: LD Plan-Achromat, ref: 415500-1614-000). Digitalized images were obtained with a Zeiss camera (5MP HD IP). In the dishes of 6-well plates, neuronal differentiation, characterized by phase contrast microscopy and with crystal violet, was determined from 20 images corresponding to 20 microscopical fields (5 × 4) taken at the center of the culture dish. The percentages of differentiated N2a cells (cells characterized by neurite outgrowth) [9] were determined. The data shown correspond to four to five independent experiments.

### 2.3. Fluorimetric Measurement of Cell Viability with the FDA Assay

N2a treated-cells were incubated for 8 min with fluorescein diacetate (FDA, Sigma-Aldrich, St Quentin-Fallavier, France) to quantify living cells [20,21]. They were then rinsed twice with phosphate buffered saline (PBS) and lysed with Tris/HCl solution containing 1% sodium dodecyl sulfate (SDS, Sigma-Aldrich). Fluorescence was measured with excitation at 485 nm and emission at 528 nm using a plate reader (Tecan Sunrise, Tecan, Lyon, France). All assays were performed with at least four independent experiments.

### 2.4. Flow Cytometric Measurement of Transmembrane Mitochondrial Potential with DiOC_6_(3)

Variations in the transmembrane mitochondrial potential (ΔΨm) were measured with 3,3′-dihexyloxacarbocyanine iodide (DiOC_6_(3)) (Thermo Fischer Scientific, Courtaboeuf, France). After different conditions of treatments, N2a cells were stained for 10 min at 37 °C with DiOC_6_(3) used at 40 nM [58]. This allows for determination of the percentage of cells with low ΔΨm, which are characterized by a decrease in green fluorescence collected through a 520/10-nm band pass filter. Flow cytometric analyses were performed on a Galaxy flow cytometer (Partec, Münster, Germany). 10,000 cells were acquired for each sample. Data were acquired with FlowMax software (Partec) and analyzed with FlowJo software (Tree Star Inc., Ashland, OR, USA). All assays were performed within at least three independent experiments.

### 2.5. Visualization of Neurite Formation by Staining with Sulforhodamine 101

N2a cells were cultured on glass slides in 6-well plates. At the end of the treatment, cells were fixed with ethanol 70% (15 min, 4 °C). After three washes with PBS, cells were incubated with Sulforhodamine 101 (SR101) at 0.5 µg/mL [59], for 30 min at 4 °C. Then, the nuclei were counterstained with Hoechst 33342 (2 µg/mL). The slides were then mounted in fluorescent mounting medium (DakoCytomation, Dako, Coppenhagen, Denmark) and stored in the dark at 4 °C until examination using a fluorescent microscope coupled to an Apotome structured illumination system (Imager M2, Zeiss). The fluorescent signals of the samples were collected with the ZEN software (Zeiss). The length of neurites was determined with Image J software (developed by Wayne Rasband, National Institutes of Health (NIH), Bethesda, MD, USA). Data shown are representative of three independent experiments.

### 2.6. Simultaneous Observation of Mitochondria and Peroxisomes by Fluorescence Microscopy: Detection of Mitochondria after Staining with MitoTracker Red and of Peroxisomes by Indirect Immunofluorescence with an Antibody Directed Against the ABCD3 Peroxisomal Transporter

In situ, the mitochondria were revealed by staining with MitoTracker Red and the peroxisomes were detected by indirect immunofluorescence with an antibody raised against the ABCD3 peroxisomal transporter [58]. To this end, cells were cultured on glass slides in 6-well plates. At the end of the treatment, cells were washed once with pre-warmed culture medium without FBS, and incubated with pre-warmed culture medium without FBS containing MitoTracker Red (Thermo Fischer Scientific; 100 nM; 30 min; 37 °C) in a humidified atmosphere containing 5% CO_2_. The cells were then washed with filtered PBS (0.2 µm) and fixed with filtered paraformaldehyde (PFA: 4%; 15 min, room temperature (RT; 22 °C)). After washing with PBS, adherent cells were permeabilized with PFS buffer ((PBS/0.05% saponin/10% FBS), 30 min, RT), and incubated with the ABCD3 rabbit polyclonal antibody (# 11523651, Pierce/Thermo Fisher Scientific, Montigny le Bretronneux, France) diluted (1/500) in PFS buffer (1 h, RT). At the end of the incubation period, cells were washed with PBS and incubated with a goat anti-rabbit antibody coupled with 488-Alexa diluted at 1/500 in PFS buffer (30 min in the dark, RT). After washing with filtered PBS, cells were counterstained with Hoechst 33342 (1 µg/mL). The slides were then mounted in fluorescent mounting medium (DakoCytomation), and stored in the dark at 4 °C until examination under a fluorescent microscope coupled with an Apotome (Imager M2, Zeiss). The fluorescent signals of the samples were collected with the ZEN software (Zeiss). Data shown are representative of three independent experiments.

### 2.7. Transmission Electron Microscopy of Mitochondria and Peroxisomes

Transmission electron microscopy (TEM) was used to simultaneously visualize peroxisomes and mitochondria [58] in N2a cells cultured for 48 h in the absence or in the presence of ODN (10^−14^ M) without FBS. In N2a cells, TEM was performed on adherent cells in culture wells. The samples were fixed for 1 h at 4 °C in 2.5% (*w/v*) glutaraldehyde diluted in cacodylate buffer (0.1 M, pH 7.4); washed in cacodylate buffer (0.1 M, pH 7.4); incubated in the dark for 1 h at 21 °C in Tris–HCl (0.05 M, pH 9.0) containing diaminobenzidine (DAB: 2.5 mg/mL) and H_2_O_2_ (10 µL/mL of a 3% solution); washed in cacodylate buffer (0.1 M, pH 7.4) for 5 min at 21 °C; post-fixed in 1% (*w/v*) osmium tetroxide diluted in cacodylate sodium (0.1 M, pH 7.4) for 1 h at 21 °C in the dark; and rinsed in cacodylate buffer (0.1 M, pH 7.4). The preparations were then dehydrated in graded ethanol solutions and embedded in Epon. Ultra-thin sections (80–82 nm) were cut with an ultramicrotome, contrasted with uranyl acetate and lead citrate, and examined using an H7500 electron microscope (Hitachi, Tokyo, Japan). One experiment was performed.

### 2.8. Evaluation of the Activation of PKA, PLC, PKC and MEK/ERK Signaling Pathways in ODN-Treated N2a Cells

To characterize the signaling pathway(s) involved in N2a cell differentiation, the cells were simultaneously treated with ODN (10^−14^ M) associated with different inhibitors H89 (20 μM), U73122 (1 μM), chelerythrine (1 μM) (Sigma-Aldrich), and U0126 (20 μM) (Calbiochem, San Diego, CA, USA), which are PKA, PLC, PKC, and MEK inhibitors, respectively. These compounds were introduced in the culture medium without FBS or containing 10% FBS, 30 min before ODN. H89 was prepared as a stock solution at 1 mM in distilled water; U0126, U73122 and chelerythrine were prepared as stock solutions in DMSO at 0.1 mM, 0.1 mM, and 1 mM, respectively. At the end of the treatment, cells were incubated for 10 min at 37 °C with 15 μg/mL FDA, rinsed twice with PBS and lysed with a Tris/HCl solution containing 1% SDS. Fluorescence was measured with excitation at 485 nm and emission at 528 nm using a Tecan Sunrise plate reader. All assays were performed in triplicate at least in four independent experiments.

### 2.9. Determination of the Fatty Acid Profile by Gas Chromatography

The lipids were extracted according to the method of Moilanen and Nikkari [60]. C19:0 was used as an internal standard. The lipids were transmethylated using boron trifluoride in methanol, in accordance with the protocol of Morrison & Smith [61]. The fatty acid methyl esters were then analyzed under the conditions described previously [62,63]. The data were processed with EZChrom Elite software (Agilent Technologies, Massy, France) and are reported in nmoles/10^6^ cells. Three independent experiments were performed.

### 2.10. Isotope Dilution Mass Spectrometry Analysis for Cholesterol and Cholesterol Precursors

Cholesterol and cholesterol precursors [46,64] were quantified on control (untreated cells) and ODN-treated cells. Cellular homogenates, prepared from pellets of 10^7^ cells suspended in water (100 μL) and sonicated for 10 min, were added to a screw-capped vial sealed with a Teflon septum, together with 200 ng D4-lathosterol, 50 ng D7-7α-hydroxycholesterol, D7-7β-hydroxycholesterol, D7-7-oxo-cholesterol, D6-Cholestane-3β,5α,6β-triol, D7-24S-hydroxycholesterol, D6-25-hydroxycholesterol and D6-27-hydroxycholesterol and 5 µg of D6-cholesterol as internal standards. To prevent auto-oxidation, 50 µL butylated hydroxytoluene (5 g/L) and 50 µL EDTA (10 g/L) were added to each vial and flushed with argon for 20 min to remove air. Alkaline hydrolysis was allowed to proceed at RT (22 °C) with magnetic stirring for 1 h in the presence of ethanolic 1 M potassium hydroxide solution. After hydrolysis, the sterols were extracted twice with 5 mL cyclohexane. Oxysterols were eluted by SPE cartridge with isopropanol: hexane 70:30. The organic solvents were evaporated under a gentle stream of argon and converted into trimethylsilyl ethers with *N,O*-bis(trimethylsilyl)trifluoroacetamide (BSTFA) with trichlorosilane (TCS) 1% (Pierce/Thermo Fisher Scientific, Illkirch, France).

Gas chromatography-mass spectrometry (GC-MS) analysis was performed on a Clarus 600D (Perkin Elmer, Waltham, MA, USA). The GC was equipped with an Elite column (30 m × 0.32 mm internal diameter (id) × 0.25 mm film; Perkin Elmer, Waltham, MA, USA), and injection was performed in splitless mode and using helium (1 mL/min) as a carrier gas. The temperature program was as follows: initial temperature of 180 °C was held for 1 min, followed by a linear ramp of 20 °C/min to 270 °C, and then a linear ramp of 5 °C/min to 290 °C, which was held for 10 min. The mass spectrometer operated in the selected ion-monitoring mode. Peak integration was performed manually, and sterols were quantified from selected-ion monitoring analyses against internal standards using standard curves for the listed sterols. Additional qualifier (characteristic fragment ions) ions were used for structural identification [65,66]. Three independent experiments were performed.

### 2.11. Statistical Analysis

Statistical analysis was performed using the GraphPad Prism5 software (San Diego, CA, USA). A Mann-Whitney test or a ANOVA test followed by a Bonferroni’s test were used. A *p* value of 0.05 or less was considered as statistically significant.

## 3. Results

### 3.1. Quantification of Neuronal Differentiation of N2a Cells Induced by ODN

**Figure 1:**
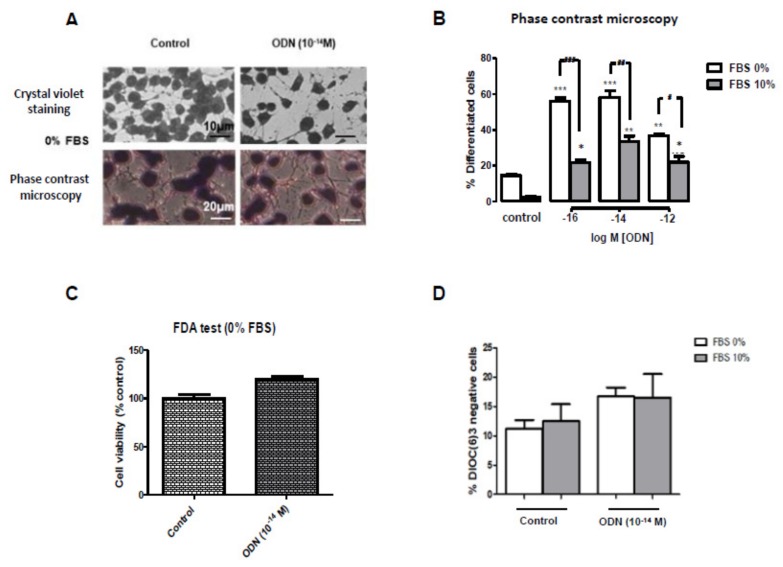
Effect of ODN on neuronal differentiation and cell viability in N2a cells. Murine neuronal N2a cells, previously cultured for 24 h in conventional culture medium, were further cultured for 48 h in medium with 10% FBS or without FBS (0% FBS) or in the presence or absence of octadecaneuropeptide (ODN: 10^−16^ to 10^−12^ M). To distinguish between undifferentiated and differentiated cells (cells characterized by neurite outgrowth), cells were either stained with crystal violet and observed by brightfield microscopy, or were directly observed by phase contrast microscopy (**A**); the percentage of differentiated cells was quantified on cells observed by phase contrast microscopy (**B**). At the concentration of ODN (10^−14^ M) inducing the highest percentage of differentiation (in the absence of FBS), the impact of ODN on cell viability was quantified by fluorimetry with the FDA assay (**C**), and by flow cytometry after staining with DiOC_6_(3) which allows to measure transmembrane mitochondrial potential (ΔΨm) (**D**). In the conditions inducing the highest percentage of differentiation (ODN 10^−14^ M; 0% FBS), different types of cells were distinguished after staining with crystal violet or by phase contrast microscopy (**A**): undifferentiated cells (without neurites); differentiated cells (neurites 5–10 µm length); differentiated cells (one or more neurites > 10 µm without or with neurites 5–10 µm length). Each value corresponds to the mean ± standard deviation (SD) of four independent experiments. ANOVA followed by Bonferroni’s test: * *p* < 0.05, ** *p* < 0.01; *** *p* < 0.001 (ODN-treated versus untreated cells); # *p* < 0.05; ## *p* < 0.01; ### *p* < 0.001 (ODN-treated cells without FBS versus ODN-treated cells with FBS).

N2a cells were cultured for 48 h in DMEM with or without 10% FBS in the presence or absence of very low concentrations (10^−16^ to 10^−12^ M) of ODN, to evaluate the ability of ODN to induce neuronal differentiation. Under these conditions, the neuronal differentiation induced by ODN was morphologically determined by neurite outgrowth (dendrites and/or axons) either by brightfield microscopy after staining with crystal violet, or by phase contrast microscopy. Data shown in Figure 1A were obtained by phase contrast microscopy from 20 images. As shown in Figure 1A, ODN (10^−14^ M) in FBS-free medium (0% FBS) increased the percentage of differentiated cells with neurites (dendrites and/or axons), and similar observations are found in the presence of 10% FBS. However, the maximum effect was observed with ODN (10^−16^ and 10^−14^ M) in the absence of FBS (Figure 1B).

Cell viability was further evaluated using the FDA assay. In FBS-free medium, no significant differences of cell viability were observed between untreated cells (control) and ODN (10^−14^ M)-treated N2a cells (Figure 1C). In addition, ODN (10^−14^ M), in the presence or absence of 10% FBS, had no effect on the percentage of cells with depolarized mitochondria (% DiOC_6_(3) negative cells) (Figure 1D).

In terms of differentiation, N2a cells were more deeply characterized. The intense blue staining of the cytoplasm of N2a cells treated with ODN (10^−14^ M) and stained with cresyl blue favors an increase in the density of Nissl bodies in the cytoplasm and constitutes a histocytological criterion of neuronal differentiation supplementing the morphological criterion (Supplementary Figure S2). In addition, in the conditions inducing the highest percentage of differentiation (ODN 10^−14^ M; 0% FBS) different types of cells were distinguished (Figure 1A): undifferentiated cells (without neurites); differentiated cells (neurites 10–20 µm length); differentiated cells (one or more neurites > 20 µm without or with neurites 10–20 µm length). High percentages of these different types of differentiated cells were present in N2a cells treated with ODN (10^−14^ M) (Figure 1A; Table 1).

Of note, the ability of ODN (10^−14^ M) to induce neuronal differentiation (neurite outgrowth) evaluated by phase contrast microscopy was also observed on human neuroblastoma SK-N-BE cells (Supplementary Figure S3).

### 3.2. Characterization of N2a Differentiated Cells after Coloration with Sulforhodamine 101 

**Figure 2:**
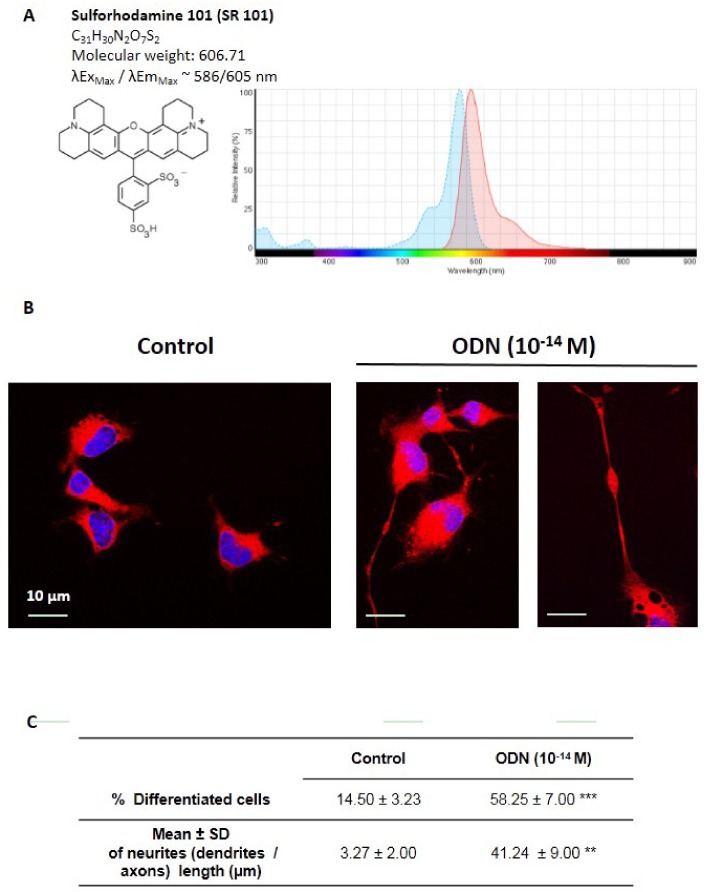
Visualization of neurites (dendrites and/or axons) in ODN-treated N2a cells. Murine neuronal N2a cells were previously cultured for 24 h in conventional cultured medium; N2a cells were further cultured for 48 h in medium without FBS in the presence or absence of octadecaneuropeptide (ODN: 10^−14^ M). Fixed cells (ethanol 70%; 15 min; 4 °C) were further stained with Sulforhodamine 101 (0.5 µg/mL; 30 min; 4 °C). The nuclei were counterstained with Hoechst 33342 (2 µg/mL). (**A**): main characteristics of Sulforhodamine 101 (SR101); (**B**): untreated cells (control); ODN-treated cells, differentiated neural cells with several neurites (long dendrites and/or axons) are observed; (**C**): Table reporting the % of N2a differentiated cells as well as the length of neurites (dendrites/axons) of differentiated cells; the values were obtained on Sulforhodamine 101 (SR101) – stained N2a cells. The images were acquired under a fluorescent microscope coupled with an Apotome (Zeiss). A Mann-Whitney test was used to compare untreated (control) and ODN-treated cells: ** *p* < 0.01; *** *p* < 0.001.

Suforhodamine 101 (SR 101) is a water-soluble, nonfixable red fluorescent polar tracer (λEx_Max_/λEm_Max_ ~ 586/605 nm) that can be used for investigating neuronal morphology. It is used to quantify protein content in whole cells [67], and is of interest in vitro on neuronal cells to distinguish between neuroblasts and neurons, to visualize the neurites, and to identify particular structures (organelles: mitochondria, peroxisomes) and antigens along the neurites (Figure 2A). The morphological characterization of neuronal differentiation of N2a cells induced by ODN was evaluated after staining with SR101. Under these conditions, compared to untreated N2a cells, ODN-treated N2a cells show higher percentages of differentiated cells with neurite outgrowth (Figure 2B). The staining assay with SR101 is therefore well adapted for the visualization and observation of neurites (dendrites and/or axons); it also permits determining the neurites (dendrites and/or axons) length, which is of 3.27 ± 2.00 µm and 41.24 ± 9.00 µm in control and ODN (10^−14^ M)-treated cells, respectively (Figure 2C). This staining procedure with SR101 brings additional evidence that ODN favors neuronal differentiation leading to neurite outgrowth in N2a cells. 

### 3.3. Effect of ODN on the Topography of Mitochondria and Peroxisomes in N2a Differentiated Cells: Evaluation by Fluorescence and Transmission Electron Microscopy

**Figure 3:**
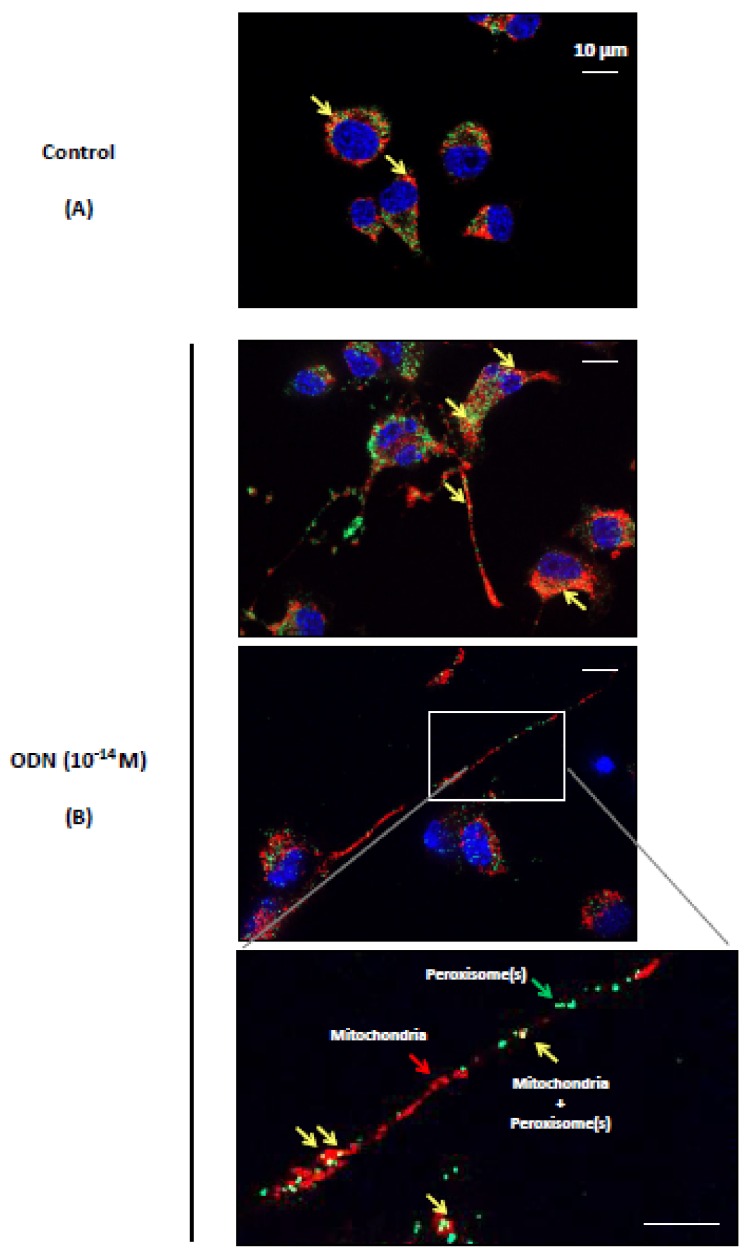
Visualization of mitochondria and peroxisomes in ODN-treated N2a cells. Murine neuronal N2a cells were previously cultured for 24 h in conventional cultured medium; the cells were further cultured for 48 h in medium without FBS in the absence (control, (**A**)) or presence of octadecaneuropeptide (ODN; 10^−14^ M, (**B**)). The mitochondria were detected with Mitotracker Red (red fluorescence) and the peroxisomes by indirect immunofluorescence after staining with an antibody raised against the ABCD3 peroxisomal transporter revealed with 488-Alexa (green fluorescence). The nuclei were counterstained with Hoechst 33342 (2 µg/mL). The images were acquired under a fluorescent microscope coupled with an Apotome (Zeiss). No neurites (dendrites and/or axons) are detected in control cells (**A**) whereas very long neurites were observed in ODN-treated cells (**B**). Along the neurites, several mitochondria (red fluorescence) and peroxisomes (green fluorescence) were detected. Yellow spots (colocalization of mitochondria and peroxisomes) were also detected. Green arrows point towards peroxisomes; red arrows point towards mitochondria; yellow arrows point towards colocalized peroxisomes and mitochondria.

**Figure 4:**
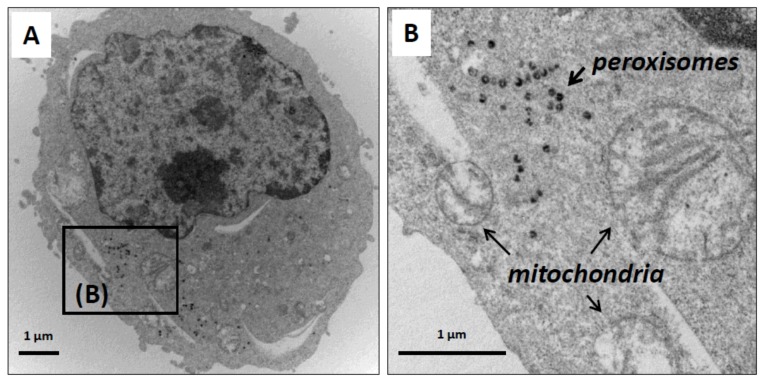
Visualisation of mitochondria and peroxisomes in untreated N2a cells by transmission electron microscopy. Murine neuronal N2a cells were previously cultured for 24 h in conventional cultured medium; the cells were further cultured for 48 h in medium without FBS. The preparations, which were contrasted with uranyl acetate and lead citrate, were also stained with DAB to visualize the peroxisomes. In untreated N2a cells, isolated peroxisomes were rarely detected in the cytoplasm whereas clusters of peroxisomes were often observed (**A**) in regions where several mitochondria were also present. The diameters of the peroxisomes were in the range of 0.1 µm (**B**).

**Figure 5:**
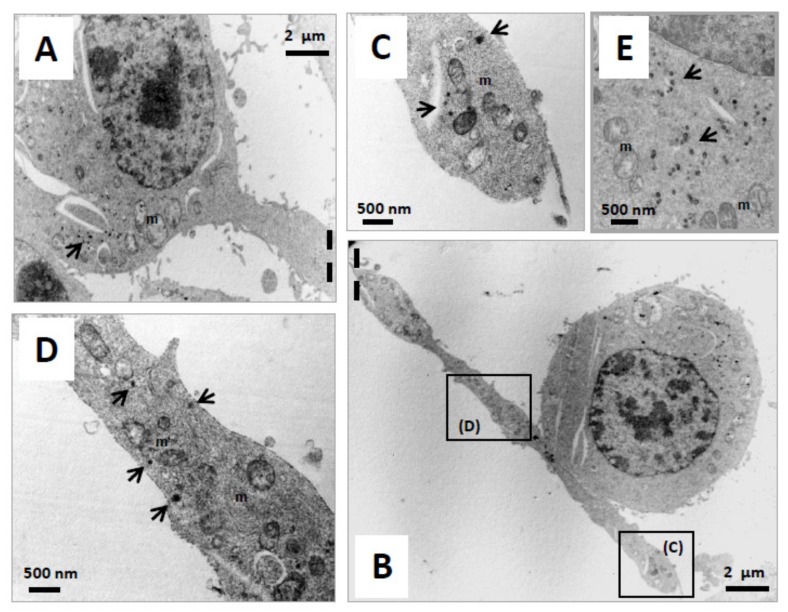
Visualization of mitochondria and peroxisomes in ODN-treated N2a cells by transmission electron microscopy. Murine neuronal N2a cells were previously cultured for 24 h in conventional cultured medium and further cultured for 48 h in medium without FBS in the presence of octadecaneuropeptide (ODN: 10^−14^ M). The preparations, which were contrasted with uranyl acetate and lead citrate, were also stained with DAB to visualize the peroxisomes. In ODN-treated N2a cells, isolated peroxisomes were rarely detected whereas clusters of peroxisomes are often observed ((**A**,**E**); **arrows**) in the soma; the diameters of the peroxisomes were in the range of 0.1 µm (**E**). Due to the large length of the neurites (dendrites/axons), these structures are often separated from the soma (cell body) and present on the grid in a compartment adjacent to the one where the corresponding soma is detected ((**A**): soma; (**B**): neurite). The simultaneous presence of mitochondria (**m**) and peroxisomes (dark arrow; diameter around 0.1 µm) was revealed in a different area of the neurite (**C**,**D**).

Mitochondria and peroxisomes are dynamic and multifunctional organelles that play pivotal roles in redox homeostasis and cell metabolism, and it is now well recognized that mitochondria and peroxisomes are tightly connected organelles [26,27,68]. At the moment, there is no information available on the impact of neuronal differentiation on the distribution of mitochondria and peroxisomes in the major cell compartments of mature neurons, and on the simultaneous status of mitochondrial and peroxisomal topography in the axon. To this end, N2a cells were cultured for 48 h in DMEM without FBS, in the presence or absence of ODN (10^−14^ M). The observations performed under a fluorescent microscope coupled with an Apotome allowing the detection of the mitochondria, which are colored in red (MitoTracker Red), and the peroxisomes, which are colored in green (ABCD3 peroxisomal transporter revealed with Alexa 488). In untreated cells, the colocalization of mitochondria and peroxysomes gives off a yellow fluorescent signal (red + green) in the cytoplasm (Figure 3A). In ODN-treated cells, mitochondria and peroxisomes were not only observed in the cytoplasm of the cell body but also in the dendrites/axons (Figure 3B). Focus on neurites (dendrites/axons) showed several areas rich in mitochondria and peroxisomes, including areas with co-localized organelles (Figure 3B). Therefore, ODN-induced neuronal differentiation is associated with neurite outgrowth. Isolated mitochondria and peroxisomes, as well as co-localized/tightly connected (mitochondria + peroxisomes) present both in the cell body (soma) as well as in the neurites (dendrites/axons) of ODN-treated cells were observed. To evaluate the size, shape, and topography of organelles (mitochondria, peroxisomes), transmission electron microscopy (TEM) was also used. Whereas mitochondria can be easily detected by TEM, the visualization of peroxisomes requires a staining procedure with DAB [69,70,71], which reveals activity of the catalase, a specific peroxisomal enzyme linked with peroxisomal β-oxidation [72]. In the present study, mitochondria and peroxisomes were simultaneously detected by TEM using the DAB staining procedure in N2a cells cultured for 48 h in FBS-free medium in the presence or absence of ODN (10^−14^ M). In untreated cells, peroxisomes were often accumulated in clusters in a particular area in the cytoplasm of the soma (Figure 4A), and they were often observed in the vicinity of mitochondria (Figure 4B). These peroxisomes are spherical homogeneous structures with a diameter of around 0.1 µm (Figure 4B). In ODN-treated cells, we focused on N2a differentiated cells which are characterized by the presence of neurites which can reach 100 µm in length. By TEM, it is therefore difficult to simultaneously observe on the same microscopical field and on the same part of the observation grid the soma and the whole neurite. In ODN-treated N2a cells, mitochondria and peroxisomes were simultaneously detected in the soma (Figure 5A,E). They were also simultaneously present at different levels of the axon including the synaptic terminal (Figure 5B–D), and their morphological characteristics were similar to those of peroxisomes of untreated cells, which are undifferentiated cells (neuroblasts), i.e., spherical structures with a diameter of around 0.1 µm. 

### 3.4. Characterization with Different Inhibitors of the Signaling Pathways Involved in the Neuronal Differentiation of ODN-Treated N2a Cells

**Figure 6:**
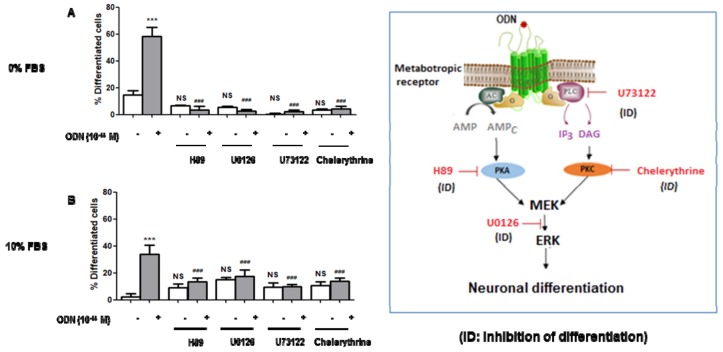
Signaling pathways triggered by ODN during neuronal differentiation of N2a cells. Murine neuronal N2a cells previously cultured for 24 h in conventional medium were further cultured for 48 h in medium without (**A**) or with 10% FBS (**B**) in the absence or in the presence of (ODN: 10^−14^ M) without or with different inhibitors: H89 (2 × 10^−5^ M; PKA inhibitor), U73122 (10^−7^ M; PLC inhibitor), chelerythrine (10^−7^ M; PKC inhibitor) and U0126 (10^−6^ M; MEK inhibitor). The percentages of differentiated cells were determined by phase contrast microscopy. ANOVA test followed by Bonferroni’s test was performed: *** *p* < 0.001 versus control; ^###^ *p* < 0.001 versus ODN-treated cells; NS (not statistically different) inhibitor versus (ODN + inhibitor).

Currently, some information is available on the signaling pathways associated with the cytoprotective activities of ODN. It is now well established that PKA/PKC/MAP kinase dependent pathways are involved [19,23]. Therefore, we asked whether similar pathways were involved in the differentiating activity of ODN on N2a cells. To this end, various inhibitors were used: H89 (20 µM), U73122 (1 µM), chelerythrine (1 µM), and U0126 (20 µM), which inhibit protein kinase A (PKA), phospholipase C (PLC), protein kinase C (PKC) and MEK, respectively. In those conditions, the differentiating properties of ODN (10^−14^ M) were inhibited by these different compounds when the cells were cultured either with or without 10% FBS (Figure 6). 

### 3.5. Effect of ODN on Fatty Acid, Cholesterol and Cholesterol Precursor Profiles in N2a Cells

**Figure 7:**
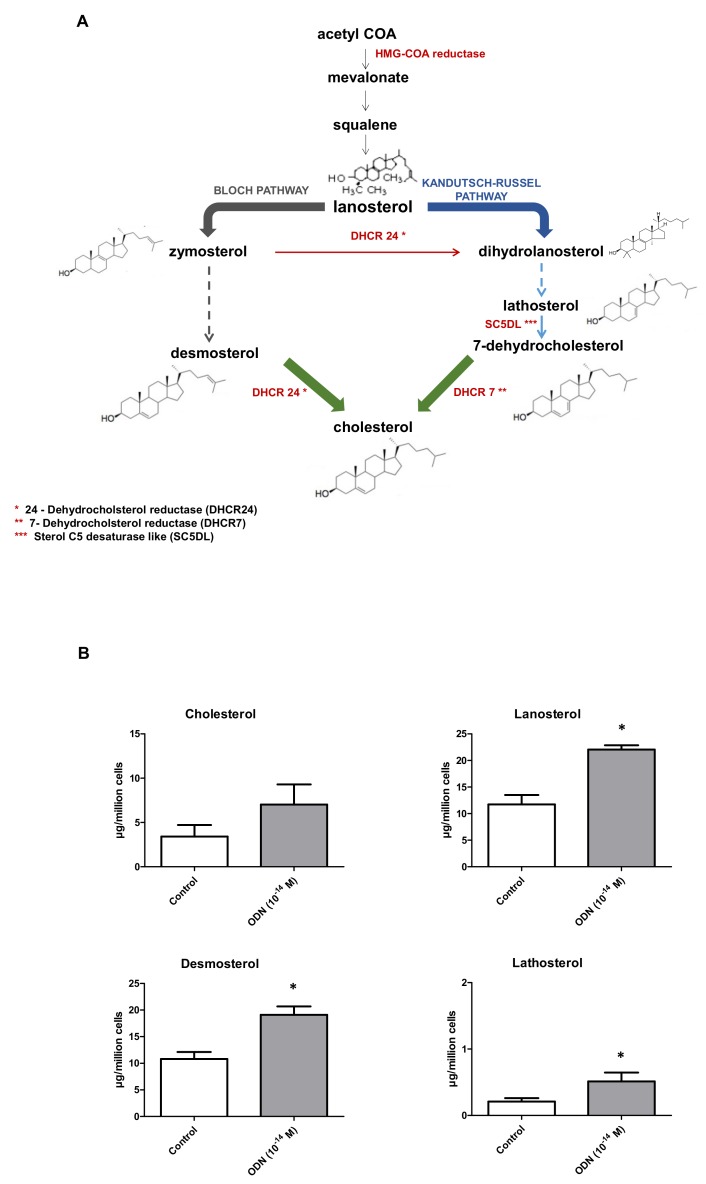
Effect of ODN on cholesterol and cholesterol precursor profiles in N2a cells. Lanosterol, the final product of the mevalonate pathway, desmosterol (specific of the Bloch pathway), and lathosterol (specific of the Kandutsch-Russel pathway) are three precursors of cholesterol (**A**). Cholesterol, lanosterol, desmosterol, and lathosterol were quantified by GC/MS in untreated and ODN (10^−14^ M) – treated N2a cells cultured without FBS (**B**). Data are expressed in µg/10^6^ cells; each value represents a mean ± SD of 3 independent experiments. Statistical analysis: Mann-Whitney test was used to compare untreated (control) and ODN-treated cells: * *p* < 0.05. No significant differences were observed between control and ODN treated cells.

It is well known that mitochondria and peroxisomes are involved in fatty acid and cholesterol metabolism [72,73,74,75]. In addition, lipid metabolism influences nerve cell proliferation and neurogenesis [76], fatty acids enhance neuronal differentiation [77], and the isoprenoid cholesterol biosynthetic pathway can be modulated during neuronal differentiation [78]. Therefore, we determined by GC the fatty acid profile (fatty acids from C12:0 to C26:1); and by GC-MS the cholesterol content and the profile of some cholesterol precursors (lanosterol, desmosterol, lathosterol) during ODN-induced neuronal differentiation on N2a cells. Compared to control cells (FBS-free medium), ODN-treated cells exhibited no statistically significant differences in terms of total fatty acids, saturated fatty acids (from C12:0 to C26:0), mono-unsaturated fatty acids (from C14:1 to C26:1), and poly-unsaturated fatty acids (from C18:2 n-6 to C24:6 n-3) (Table 2). Whereas not statistically significant, the cholesterol content per cell was higher in ODN-treated cells than in control; however, main cholesterol precursors (lanosterol, desmosterol (belonging to the Bloch pathway), lathosterol (belonging to the Kandutsch-Russel pathway)) were significantly higher in ODN-treated cells than in control (Figure 7). 

## 4. Discussion

In the present study, we demonstrate that the neuropeptide ODN induces neuronal differentiation of murine N2a cells that is extensively used to study neuronal differentiation, neurite growth, and the associated signaling pathways. This differentiation process triggers a PKA/PLC/PKC/MEK/ERK-dependent signaling pathway which is also required in ODN-induced cytoprotection [22]. In addition, our data show that ODN-induced neuritogenesis is associated with dynamic topographical modifications of mitochondria and peroxisome, and an increase in cholesterol and cholesterol precursor biosynthesis.

The neuropeptide ODN is highly expressed in the developing nervous system [22,79] and has been shown to exert a potent cytoprotective and antioxidant effects in cultured murine astrocytes and cerebellar granule neurons in vitro [18,19,23] and dopaminergic neurons in vivo [21]. Data in the literature indicate that neuropeptides such as BDNF, and nerve growth factor (NGF) exhibiting cytoprotective activities, may also induce cell differentiation [12,80]. As in the case of ODN, the neuropeptide PACAP, which protects both neurons and glial cells against oxidative stress [81,82], also induces PC12 cell differentiation associated with neurite outgrowth [53,54]. It is noteworthy that the effect of ODN on the growth of dendrites and axons is observed at a very low concentration, 10^−14^ M both in FBS-free medium and that containing 10% FBS. Consistent with this observation, in vivo and in vitro studies have shown the capacity of ODN to prevent oxidative damage-induced neuronal cell death at very low concentrations, i.e., within the fentomolar range, with no additional protective effect at higher concentrations [19,21]. Neuronal differentiation and protection from apoptosis have also been observed with other femtomolar-acting compounds from astroglial cells such as activity-dependent neuroprotective protein (ADNP) and ADNP-derived peptide (NAP), and activity-dependent neurotrophic factor (ADNF) [83,84]. Thus, the present data identify ODN as a new player of this growing family of femtomolar-acting gliopeptides that exhibit both potent neuroprotective and differentiating activities. Compared to retinoic acid and polyphenols (apigenin, resveratrol) which have been reported to induce neuronal differentiation of N2a cells at micromolar concentrations [9], one of the main advantages of ODN comparatively to retinoic acid and polyphenols, mainly resveratrol, is to trigger differentiation without cytotoxic effects (loss of plasma membrane integrity; loss of transmembrane mitochondrial potential (ΔΨm)). Indeed, ODN (10^−14^ M) induces cell differentiation of N2a cells without cell death, evaluated with FDA and DiOC_6_(3). Previous kinetic and doses-responses studies revealed that, even at higher doses (in the range 10^−12^ M to 10^−8^ M), ODN is devoid of cytotoxic effects whatever the time or the dose [17,20,23], indicating that the gliopeptide ODN did not exhibit by itself toxic effects on N2a cells. However, it cannot be excluded that the decrease in the responses of N2a cells to high concentrations of ODN might in part be due to desensitization of its receptors and/or the proteolytic breakdown of the peptide. As neuronal cells acquire excitability and start to express some genes that provide their functional identity [85], we can also suppose that cytotoxic effects might interfere with gene expression.

To define the signaling pathway associated with the differentiating effect of ODN, different inhibitors linked to the cytoprotective signaling pathway of ODN were used: H89 (PKA inhibitor), U73122 (PLC inhibitor), chelerythrine (PKC inhibitor) and U0126 (MEK inhibitor) [22]. Interestingly, these different molecules, which inhibit the cytoprotective activity of ODN, are also able to prevent its differentiating activity. Moreover, it has been reported by using quantitative western blot analysis, that ODN, at very low doses, stimulates ERK1/2 phosphorylation, and that PKA, PKC, and MEK blockers abrogates the stimulatory action of ODN, as well as its effect on cell protection, both in astroglial cells and neurons [19,23]. These data show that the cytoprotection and differentiation induced by the ODN have common signaling pathways. In these two processes, the PKA, PKC, MEK-ERK pathway is activated. It is widely accepted that signaling events downstream of PKA, and PKC/ERK1/2 activation lead to Akt phosphorylation in various cell types [86], and that sustained activation of MAPK and Akt, similar to the one observed with ODN, promotes neuronal survival [22,87], suggesting that PI3K/Akt signaling cascade may contribute to the effect of ODN on neuroprotection but also on the differentiation of N2a cells and may thus contribute to the cytoprotective and trophic effect of ODN. In addition, the involvement of PKA/PLC/PKC/ERK kinases in the effect of ODN on neuroprotection/differentiation of N2a cells is supported by previous studies. We have previously shown that the use of a cell-permeant cAMP analog, dbcAMP, or the use of forskolin, an activator of adenylate cyclase, mimicks the protective effects of ODN upon the deleterious action of oxidative stress on astrocytes [23]. Furthermore, Algarni et al. [88] already reported that activation of PKA and PKC pathways promote N2a survival. Altogether, these data brings additional evidences supporting that the PKA/PLC/PKC/ERK kinases are involved in the effects of ODN on the neuroprotection/differentiation process on N2a cells. As the growth of neurites (dendrites and/or neurons) requires much energy and mitochondrial biogenesis [89], the impact of ODN on mitochondrial and peroxisomal topography was also studied. Indeed, in well-differentiated neuronal cells, a topographical redistribution of mitochondria throughout the axon is needed to privilege the transmission of the nerve impulse [90]. The present data show important topographical changes of the mitochondria in the soma of N2a cells cultured in the presence of ODN, and they also reveal several mitochondria in the neurites (dendrites and/or axons). It is also well known that mitochondria also play a key role in lipid metabolism (fatty acids, cholesterol) which is required for the biogenesis the plasma membrane of dendrites and axons [91]. In addition, it is well known that long chain polyunsaturated fatty acids (C < 22), which are metabolized by the mitochondria, and are essential for the growth and development of neurons [92]. However, no significant difference in the fatty acid profile was observed between the control and ODN-treated cells. However, as higher levels of cholesterol and of some of its precursors (lanosterol, desmosterol, lathostererol) are found, this suggest important modifications of cholesterol metabolism and of plasma membrane composition in ODN-treated cells. These observations support that the fatty acid profile is similar in undifferentiated and differentiated neuronal cells. In contrast, as differences in the levels of cholesterol and cholesterol precursors are observed between undifferentiated and differentiated cells, our data suggest differences in cholesterol metabolism in neuroblasts and mature neurons.

Our data also show that in the presence of ODN, important topographical changes of peroxisomes in the soma of N2a cells occur, and they also reveal several peroxisomes, sometimes associated with mitochondria, in the neurites (dendrites and/or axons). Currently, there are multiple lines of evidence that mitochondria and peroxisomes are tightly connected. Physical and functional interactions have been described between these two organelles [27], and the analysis of the communication between mitochondria and peroxisome has recently emerged [26]. It is also well recognized that the mitochondria and peroxisomes have cooperative roles in the metabolism of cellular lipids (fatty acids, cholesterol) and reactive oxygen species [68]. Moreover, it is well established that peroxisome movement, distribution, and interactions are essential for cell activity [26,93]. The present results agree with a dynamic spatial adaptation and organization of peroxisomes, which allows the movement of these organelles into areas such as dendrites and/or axons to assume the metabolic needs associated with neurotransmission. This also supports that environmental changes, in this case the presence of the gliopeptide ODN, can influence the topographical repartition of peroxisomes in the soma, and neurites of N2a cells [94]. It is also known that the peroxisome plays key role in lipid metabolism, and especially contributes to the beta-oxidation of VLCFA and branched chain fatty acids (e.g., pristanic acid), to the alpha-oxidation of phytanic acid and to the biosynthesis of ether phospholipids (plasmalogens) [70,72,75,92]. The fact that ODN does not induce changes of the profile of VLCFA favors the hypothesis that the beta-oxidation of VLCFA is similar in neuroblasts and in neurons. It is therefore suggested that nerve cell differentiation is not associated with modifications of peroxisomal metabolism, mainly VLCFA metabolism.

## 5. Conclusions

The present data show that ODN, at subnanomolar concentrations (10^−14^ M), has the capacity to differentiate murine N2a cells from neuroblasts to neurons. Thus, in addition to its cytoprotective activities, mainly antioxidant properties on nerve models [18,22], ODN is able to induce neuronal differentiation of murine N2a cells characterized by neurite outgrowth, indicating that ODN can be considered a neurotrophic factor. This neuronal differentiation, which triggers a PKA/PLC/PKC/MEK-ERK signaling pathway, is also associated with a redistribution of organelles (peroxisomes and mitochondria) all along the dendrites and/or axons. As we report that the ODN also induces the differentiation of human SK-N-BE neuronal cells, this data reinforces the interest of these neuropeptide for the prevention and/or the treatment of neurodegenerative diseases associated with neuronal loss such as Alzheimer’s disease and Parkinson’s disease. Altogether, our data bring new evidence that ODN has therapeutic potential for the treatment of cerebral injuries associated with neuronal damage.

## Figures and Tables

**Table 1:** Percentages of undifferentiated and differentiated N2a cells in control and ODN-treated cells. Murine neuronal N2a cells, previously cultured for 24 h in conventional cultured medium, were further cultured for 48 h in medium without FBS in the absence or presence of octadecaneuropeptide (ODN 10–14 M). In these conditions inducing the highest percentage of differentiation (ODN 10–14 M; 0% FBS) different types of cells were distinguished: undifferentiated cells (without neurites); differentiated cells (neurites 5–10 μm length); differentiated cells (one or more neurites > 10 μm without or with neurites 5–10 μm length). The percentages of undifferentiated and differentiated cells were determined; each value are mean ± standard deviation (SD) of 3 independent experiments.

	Control (0% FBS)	ODN (10^−14^ M)–0% FBS
Undifferentiated cells (without neurites)	87.8 ± 3.5%	35.2 ± 4.2%
Differentiated cells (neurites 5–10 µm length)	4.6 ± 2.8%	30.4 ± 5.3%
Differentiated cells (one or more neurites > 10 µm length without or with neurites 5–10 µm length)	8.6 ± 4.1%	34.4 ± 3.3%

**Table 2:** Murine neuronal N2a cells, previously cultured for 24 h in conventional cultured medium, were further cultured for 48 h in medium without FBS in the presence or absence of octadecaneuropeptide (ODN 10^−14^ M). The level of long chain fatty acids (LCFA: C12 < LCFA < C22) and very long chain fatty acids (VLCFA: ≥ C22) was determined by GC/MS. Data are expressed in nmoles/10^6^ cells, and each value are mean ± standard deviation (SD) of 3 independent experiments. Statistical analysis: ANOVA followed by Bonferroni’s test: * *p* < 0.05; ODN-treated cells versus untreated cells (control). No significant differences were observed between control and ODN treated cells (Mann-Whitney test).

	Treatments	
	Control	ODN (10^−14^ M)
**Saturated fatty acids**		
**C12:0**	0.57 ± 0.34	0.67 ± 0.15
**C14:0**	6.04 ± 3.40	8.22 ± 6.00
**C15:0**	0.33 ± 0.19	0.40 ± 0.24
**C16:0**	37.95 ± 20.47	47.50 ± 27.36
**C17:0**	0.28 ± 0.14	0.33 ± 0.16
**C18:0**	19.40 ± 10.27	25.23 ± 15.70
**C19:0**	0.03 ± 0.02	0.04 ± 0.02
**C20:0**	0.34 ± 0.18	0.47 ± 0.31
**C21:0**	0.01 ± 0.01	0.01 ± 0.01
**C22:0**	0.07 ± 0.05	0.10 ± 0.07
**C23:0**	0.01 ± 0.01	0.01 ± 0.01
**C24:0**	0.22 ± 0.13	0.28 ± 0.21
**C25:0**	0.01 ± 0.01	0.02 ± 0.01
**C26:0**	0.10 ± 0.06	0.12 ± 0.09
**Mono-unsaturated fatty acids**		
**C14:1**	0.02 ± 0.01	0.03 ± 0.01
**C16:1 n-7**	0.94 ± 0.56	1.28 ± 0.95
**C16:1 n-9**	0.79 ± 0.46	1.05 ± 0.70
**C18:1 n-7**	10.75 ± 6.48	15.39 ± 12.95
**C18:1 n-9**	54.64 ± 25.64	69.53 ± 37.16
**C20:1 n-7**	0.71 ± 0.37	1.10 ± 0.90
**C20:1 n-9**	3.00 ± 1.66	4.78 ± 4.09
**C22:1 n-7**	0.06 ± 0.03	0.09 ± 0.07
**C22:1 n-9**	0.45 ± 0.27	0.68 ± 0.58
**C24:1 n-9**	0.26 ± 0.16	0.37 ± 0.29
**C26:1**	0.11 ± 0.06	0.14 ± 0.10
**Poly-unsaturated fatty acids**		
**C18:2 n-6**	0.79 ± 0.47	1.04 ± 0.80
**C18:3 n-3**	0.01 ± 0.01	0.02 ± 0.01
**C18:3 n-6**	0.04 ± 0.02	0.05 ± 0.03
**C20:2 n-6**	1.17 ± 0.53	1.79 ± 1.24
**C20:3 n-6**	0.45 ± 0.24	0.60 ± 0.40
**C20:3 n-9**	3.42 ± 1.73	5.02 ± 3.64
**C20:4 n-6**	1.97 ± 1.29	2.40 ± 1.51
**C20:5 n-3**	0.42 ± 0.27	0.58 ± 0.51
**C22:2 n-6**	0.75 ± 0.37	1.08 ± 0.74
**C22:4 n-6**	0.21 ± 0.20	0.26 ± 0.18
**C22:5 n-3**	0.83 ± 0.56	0.98 ± 0.57
**C22:5 n-6**	0.01 ± 0.01	0.02 ± 0.01
**C22:6 n-3**	1.37 ± 0.90	1.68 ± 1.07
**C24:4**	0.00 ± 0.01	0.01 ± 0.01
**C24:5**	0.01 ± 0.01	0.01 ± 0.01
**C24:6 n-3**	0.08 ± 0.05	0.10 ± 0.08
**Total fatty acids**	148.62 ± 10.55	192.48 ± 13.28

## Supplementary Materials

The following are available online.
