# Supplementary material for: Octadecaneuropeptide (ODN) Induces N2a Cells Differentiation through a PKA/PLC/PKC/MEK/ERK-Dependent Pathway: Incidence on Peroxisome, Mitochondria, and Lipid Profiles
Source: Molecules. 2019 Sep 11;24(18):3310. doi: 10.3390/molecules24183310 (PMC6767053; doi:10.3390/molecules24183310)
Supplement: Supplementary file 1 [file molecules-24-03310-s001.pdf]

Octadecaneuropeptide (ODN) Induces N2a Cells Differentiation through a PKA/PLC/PKC/MEK/ERK-Dependent Pathway: Incidence on Peroxisome, Mitochondria, and Lipid Profiles

Amira Namsi [1,2], Thomas Nury [1], Amira. S. Khan [3], Jérôme Leprince [4,5], David Vaudry [4,5], Claudio Caccia [6], Valerio Leoni [7], Atanas G. Atanasov [8,9,10], Marie-Christine Tonon [4], Olfa Masmoudi-Kouki [2,*] and Gérard Lizard [1,*]

1 Team Bio-PeroxIL, Biochemistry of the Peroxisome, Inflammation and Lipid Metabolism (EA7270)/University Bourgogne Franche-Comté (UBFC)/Inserm, 21000 Dijon, France; amira.namsi@gmail.com (A.M.); thomas.nury@u-bourgogne.fr (T.N.)

2 Faculty of Science of Tunis, University Tunis El Manar, LR18ES03, Laboratory of Neurophysiology, Cellular Physiopathology and Biomolecules Valorisation, Tunis 2092, Tunisia

3 Physiology of Nutrition & Toxicology (NUTox), Inserm U1231, University UBFC, 21000 Dijon, France; amira.khan@u-bourgogne.fr

4 UNIROUEN, Inserm U1239, Laboratory of Neuronal and Neuroendocrine Communication and Differentiation, Normandie University, 76000 Rouen, France; jerome.leprince@univ-rouen.fr (J.L.); david.vaudry@univ-rouen.fr (D.V.); marie-christine.tonon@univ-rouen.fr (M.-C.T.)

5 UNIROUEN, Regional Cell Imaging Platform of Normandy (PRIMACEN), Normandie University,
76000 Rouen, France

6 Laboratory of Medical Genetics and Neurogenetics, Foundation IRCCS Istituto Neurologico Carlo Besta, 20100 Milan, Italy; claudio.caccia@istituto-besta.it (C.C.)

7 Laboratory of Clinical Chemistry, Hospital of Varese, ASST-Settelaghi, 20100 Milan, Italy; valerioleoni@hotmail.com (V.L.)

8 Institute of Genetics and Animal Breeding of the Polish Academy of Sciences, Jastrzebiec, 05-552 Magdalenka, Poland; atanas.atanasov@univie.ac.at (A.G.A.)

9 Department of Pharmacognosy, University of Vienna, 1010 Vienna, Austria

10 Institute of Neurobiology, Bulgarian Academy of Sciences, 23 Acad. G. Bonchev str., 1113 Sofia, Bulgaria

* Correspondence to: olfa.masmoudi@fst.utm.tn (O.M.-K.); gerard.lizard@u-bourgogne.fr (G.L.); Tel.: +216 23-06-07-34 (O.M.-K.); +33-3-80-39-62-56 (G.L.); Fax: +216-71-87-16-66 (O.M.-K.); +33-3-80-39-62-50 (G.L.)

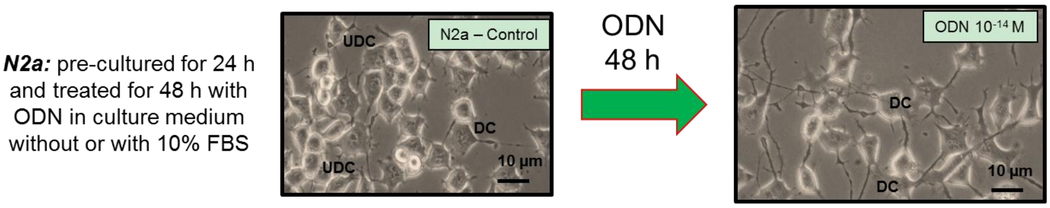

N2a cells expressing functionnal octadecaneuropeptide (ODN) receptor(s) have the ability to differentiate in neurons with neurites (dendrites and / or axons) of different length when incubated with ODN.
UDC: undifferentiated cells; DC: differentiated cells

Supplementary Figure S1: Evaluation of neuronal differentiation of N2a cells with morphological criteria by phase contrast microscopy. N2a cells (morphologically resembling neuroblasts) have the ability to differentiate in young immature and mature neurons with neurites (evocating dendrites and/or axons). Conventional morphological criteria, which were previously described [9], were used to evaluate neuronal differentiation. In control cells, mainly neuroblasts were present (cells without neurites: undifferentiated cells (UDC)). In ODN (10^{-14} M)-treated N2a cells, several differentiated cells (DC) were observed: cells with neurites of different length (average length (5-10 µm); cells with one or more neurites of important length (> 10 µm) evocating dendrites and/or axons; these neuites of important length can be associated or not with dendrites (5-10 µm length). Images were realized in phase contrast microscopy.

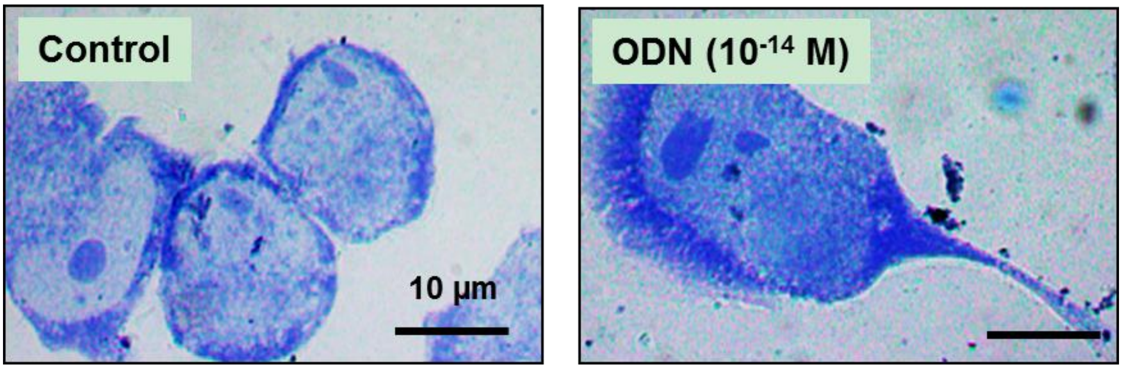

Supplementary Figure S2: Evaluation of neuronal differentiation of N2a cells after staining with cresyl blue. Cresyl blue is a conventional histocytological staining method which permits to detect Nissl bodies characteristic of neuronal cells. The intensity of the blue staining increases during neuronal differentiation: the cytoplasmic density of Nissl bodies is enhanced. Whereas a weak staining was observed in control cells, a strong blue staining was observed in ODN (10^{-14} M)-treated cells without FBS. The observations were realized in brightfield microscopy.

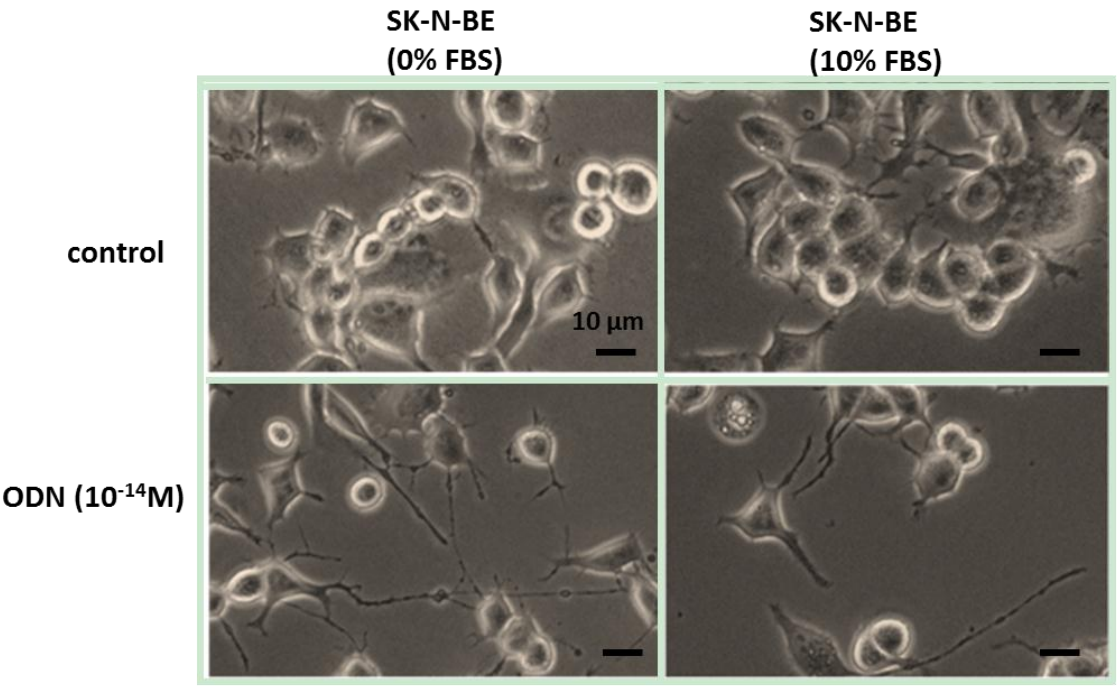

Assays	% Differentiated SK-N-BE (0% FBS)	% Differentiated SK-N-BE (10% FBS)
Control	18.73 ± 6.36	9.20 ± 5.81
ODN (10^{-14} M)	50.69 ± 3.49 **	66.19 ± 4.77 **

Supplementary Figure S3: Induction of neuronal differentiation of human SK-N-BE cells by ODN. SK-N-BE cells previously cultured in conventional medium for 24 h were further incubated for 48 h in medium without (0% FBS) or with 10% FBS in the presence or absence of ODN (10^{-14} M). Differentiated cells were characterized by neurite outgrowth. The percentage of differentiated cells was quantified from images taken under a phase contrast microscope under similar conditions than those used for N2a cells. Each value shows the mean ± SD of 5 independent experiments. Statistical analysis was performed by one-way ANOVA followed by the Bonferroni's test. ** $p < 0.01$ compared to the control.
